# Enhancing serious illness communication using artificial intelligence

**DOI:** 10.1038/s41746-022-00556-2

**Published:** 2022-01-27

**Authors:** Isaac S. Chua, Christine S. Ritchie, David W. Bates

**Affiliations:** 1 Division of General Internal Medicine and Primary Care, Department of Medicine, Brigham and Women’s Hospital, Boston, MA USA; 2 Division of Palliative Care, Department of Psychosocial Oncology and Palliative Care, Dana-Farber Cancer Institute, Boston, MA USA; 3 Harvard Medical School, Boston, MA USA; 4 Division of Palliative Care and Geriatric Medicine, Department of Medicine, Massachusetts General Hospital, Boston, MA USA

**Keywords:** Geriatrics, Quality of life, Machine learning

## Abstract

Delivery of serious illness communication (SIC) is necessary to ensure that all seriously ill patients receive goal-concordant care. However, the current SIC delivery process contains barriers that prevent the delivery of timely and effective SIC. In this paper, we describe the current bottlenecks of the traditional SIC workflow and explore how a hybrid artificial intelligence-human workflow may improve the efficiency and effectiveness of SIC delivery in busy practice settings.

Serious illness communication (SIC) is an essential component of palliative care that ensures the delivery of goal-concordant care. SIC is often defined as the conversations between clinicians and patients with serious illness about their goals, values, and priorities^1^. High-quality and timely SIC enables and enhances decision-making and care planning through the process of cultivating patients’ prognostic awareness and translating their values and priorities into patient-centered recommendations. The iterative and non-linear process of SIC requires frequent and early conversations to ensure that clinicians accurately understand patients’ evolving goals, values, and priorities and to make patient-centered recommendations throughout the illness trajectory.

The traditional SIC delivery process consists of a series of conversations where gathering, interpreting, and integrating SIC data occur within a clinical encounter followed by manual clinician documentation in the electronic health record (EHR) post-visit. This process can be broken down into the following steps: determining patient eligibility for SIC; gathering and interpreting information (e.g., eliciting and clarifying the patient’s illness understanding, hopes, and worries); conducting a therapeutic conversation (e.g., counseling and supporting the patient on coping with life-threatening illness) with the goal of shared decision-making; documenting the conversation; and making SIC documentation accessible to others in the EHR (Fig. 1). However, each step is a potential bottleneck because the ability to initiate SIC or make forward progress depends heavily on the clinician’s ability, skill, and judgement. This is problematic for several reasons.

**Fig. 1:**
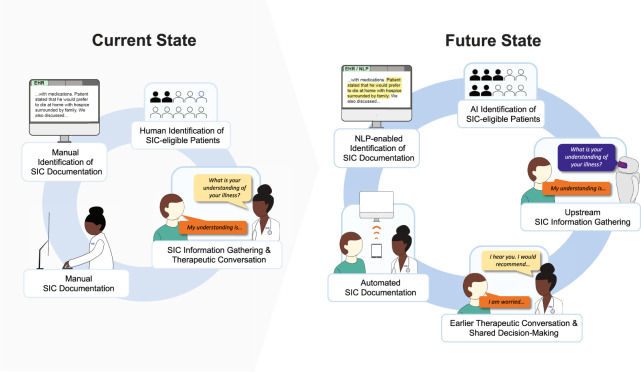
Human versus hybrid artificial intelligence (AI)-human workflow for serious illness communication (SIC). The current workflow relies on human judgment to identify SIC-eligible patients and manual effort to initiate SIC, to document SIC, and to locate SIC documentation in the electronic health record (EHR). A hybrid AI-human workflow would leverage AI to identify SIC-eligible patients more accurately and to streamline the workflow by helping complete essential menial tasks, thus ensuring more seriously ill patients will receive timely SIC and allowing clinicians more time and energy to focus on the higher-order cognitive and emotional tasks, including problem-solving. Natacha Meyer designed and illustrated the figure and provided permission to use this figure in the manuscript.

First, most clinicians lack SIC training and feel unprepared to have these difficult conversations with their patients^2^. Second, patients and/or clinicians may be unclear about the optimal timing and when to make such conversations a priority^2^. Third, clinicians often lack time to conduct SIC^2^ and to document these conversations adequately^3^. Fourth, standards for EHRs to facilitate consistent, accurate documentation that is easily accessible to all care team members are lacking^4^. Therefore, in addition to training more clinicians to be competent in SIC, a novel workflow that addresses these barriers will be necessary to ensure that all seriously ill patients receive timely and effective SIC that informs their care in real time and naturally results in documentation of patients’ goals and preferences that is visible to others. We propose that a hybrid artificial intelligence (AI)-human workflow can improve this process by helping clinicians identify patients with SIC needs more accurately; promoting upstream data collection to facilitate more efficient in-person shared decision-making; reducing clinician documentation burden by streamlining the SIC documentation process; facilitating seamless sharing of patient goals and preferences via accurate and efficient identification of SIC documentation in the EHR; and providing real-time feedback to clinicians on their SIC skills.

Patients with serious illness often experience delayed SIC because clinicians are poor at prognosticating life expectancy for terminally ill patients, usually erring on the side of optimism^5^. Moreover, systematic methods to identify patients with palliative care needs are lacking^6^. To solve this problem, AI researchers have developed machine learning algorithms to generate more accurate mortality predictions to facilitate earlier SIC and palliative care delivery^7,8^. Some researchers have demonstrated that coupling AI-generated mortality predictions with behavioral nudges to clinicians can improve SIC frequency^9^. However, critics have expressed worry about using mortality predictions alone for identifying populations with palliative care needs because a reductionistic interpretation of these results may lead to further propagation of algorithmic or other systemic biases leading to inequitable care and patient harm^10^. Therefore, others have suggested alternative metrics that identify patients at risk of worsening serious illness to train predictive algorithms—including functional decline, deteriorating quality of life, escalating caregiver burden, or psychosocial or spiritual distress^10^. Some accountable care organizations are already using claims-based algorithms to identify high-cost patients who would benefit from earlier palliative care^11^, but greater effort is needed to mitigate algorithmic bias, especially among commercially available products that are widely used^12^. Moreover, additional methods to identify SIC-eligible patients should be considered since EHR-based algorithms often have performance gaps^13^.

The use of conversational agents (aka chatbots) has largely been unexplored in palliative care. Conversational agents that are emotionally aware or use unconstrained natural language input are nascent in health care^14–16^, but the technology to date is mature enough to support its use in SIC as a basic data-gathering agent. One could imagine SIC conversational agents that would collect information about the patient’s prognostic awareness and priorities prior to in-person visits. Doing so would enable clinicians to maximize face-to-face time on higher-order cognitive and emotional tasks (e.g., interpreting patient preferences and responding empathically to a patient’s emotional state) that would lead to earlier shared decision-making. Conversational agents may also give patients time to reflect and discuss issues with trusted persons prior to meeting with the clinician.

To date, no studies on conversational agents have been conducted on patients with serious illness, but proof-of-concept studies in the general population have demonstrated the acceptability of conversational agents that address palliative care-related topics^17,18^. In one study, older adults utilized multiple-choice responses to converse with an agent that provided spiritual counseling, which reduced anxiety and increased the intent to create a last will and testament^17^. In another, machine learning algorithms allowed the agent to collect patient-reported outcome measures and display empathy to the users’ free text responses^18^. Although conversational agents were well-received in these preliminary studies, some patients will prefer to have the entire SIC with their clinicians directly, obviating the need for a chatbot. Further studies need to be conducted on actual patients with serious illness and should assess if conversational agent-led SIC triggers emotional distress in patients or actually enhances the patient-clinician relationship. Moreover, deploying conversational agents may inadvertently widen inequities in certain populations, particularly patients with limited English proficiency, health information technology literacy, or broadband access.

AI can also streamline the SIC documentation process and potentially improve the quality of SIC documentation via natural language processing (NLP)—a form of machine learning designed to understand, interpret, or manipulate human language. Missing or incomplete documentation in the EHR regarding patient preferences for life-sustaining treatment is common and contributes to medical errors related to end-of-life care^3^. NLP-enabled dictation software has demonstrated the ability to reduce medical documentation time while maintaining documentation quality^19^ and is already commercially available^20^. Such technology would reduce the time clinicians spend manually writing notes and minimize recall bias since the content of the conversation is transcribed verbatim during the conversation and not hours later, typical of much documentation. As a result, nuanced details of the conversation are readily captured in real time leading to higher quality notes with less clinician effort.

NLP also has the potential to address barriers resulting from poor EHR design that prevent or inhibit the extraction and flow of meaningful advanced care planning information across the care continuum^4^. In its current state, identifying SIC documentation in the EHR typically involves a manual chart review that possibly includes a keyword search or utilization of note filters. NLP-enabled software that identifies free text SIC documentation would likely reduce the time and effort clinicians spend looking for this information and prevent inadvertent oversight of patient preferences leading to goal-discordant care. AI-assisted chart reviews have demonstrated higher accuracy and shorter time for extracting relevant patient information compared with standard chart reviews^21^. Additionally, NLP has demonstrated the ability to identify SIC documentation accurately from EHR data and, in some cases, more accurately than human coders^22–24^. However, the accuracy of NLP to identify SIC documentation largely depends on the quality of the gold standard dataset created by human annotation used to train the model. Consequently, widespread implementation of NLP-enabled software to identify SIC documentation likely remain years away since high-quality annotated examples to train generalizable models are lacking, and adapting NLP models between different datasets often require additional training or fine-tuning^25^. In the interim, some health systems have created a centralized location for SIC documentation in the EHR to improve SIC documentation identification^9,26^, but compliance with utilizing these modules will likely remain an issue and additional NLP assistance will optimize the identification of SIC documentation in the EHR^27^.

Finally, AI has the potential to improve SIC delivery by providing speech analysis and personalized feedback to clinicians regarding their communication skills^28^. Automated speech analysis and communication feedback will likely take years to manifest because not only do technical and logistical barriers remain (e.g., lack of adequate high-quality SIC recordings to accurately assess non-linguistic features)^28^, but also greater consensus is needed to define and measure basic communication quality and outcomes^29^. Researchers are currently utilizing NLP to analyze audio recordings of SIC to characterize and understand the naturally occurring features of these complex conversations^30–32^, such as identifying intentional pauses that foster empathy, compassion, and understanding, aka “Connectional Silences.^30^" This type of research will guide future efforts to develop ways of automating the measurement of SIC quality in real time, allowing for immediate feedback to improve clinician performance.

In conclusion, a hybrid AI-human SIC workflow may improve the efficiency and effectiveness of SIC delivery in busy practice settings. Some of the AI technology are available for widespread use presently (e.g., risk prediction algorithms and NLP-enabled transcription software), whereas others are emerging technologies that are being developed and studied (e.g., SIC conversational agents and NLP-enabled identification of SIC documentation). This proposed paradigm still requires that clinicians undergo some SIC training to capitalize on the assistance provided by AI, as well as additional research to avoid unintended consequences of AI implementation. That said, a semi-automated approach to SIC delivery holds tremendous promise and would likely improve current SIC workflow by optimizing clinical manpower and efficiency while increasing the likelihood that these critically important conversations will occur effectively and in a timely fashion.
